# Local versus Generalized Phenotypes in Two Sympatric *Aurelia* Species: Understanding Jellyfish Ecology Using Genetics and Morphometrics

**DOI:** 10.1371/journal.pone.0156588

**Published:** 2016-06-22

**Authors:** Luciano M. Chiaverano, Keith W. Bayha, William M. Graham

**Affiliations:** 1 Department of Marine Science, University of Southern Mississippi, Stennis Space Center, Mississippi, United States of America; 2 American Association for the Advancement of Science, Washington, DC, United States of America; Australian Museum, AUSTRALIA

## Abstract

For individuals living in environmentally heterogeneous environments, a key component for adaptation and persistence is the extent of phenotypic differentiation in response to local environmental conditions. In order to determine the extent of environmentally induced morphological variation in a natural population distributed along environmental gradients, it is necessary to account for potential genetic differences contributing to morphological differentiation. In this study, we set out to quantify geographic morphological variation in the moon jellyfish *Aurelia* exposed at the extremes of a latitudinal environmental gradient in the Gulf of Mexico (GoM). We used morphological data based on 28 characters, and genetic data taken from mitochondrial cytochrome oxidase I (*COI*) and nuclear internal transcribed spacer 1 (*ITS-1*). Molecular analyses revealed the presence of two genetically distinct species of *Aurelia* co-occurring in the GoM: *Aurelia* sp. 9 and *Aurelia* c.f. sp. 2, named for its divergence from (for COI) and similarity to (for *ITS-1*) *Aurelia* sp. 2 (Brazil). Neither species exhibited significant population genetic structure between the Northern and the Southeastern Gulf of Mexico; however, they differed greatly in the degree of geographic morphological variation. The morphology of *Aurelia* sp. 9 exhibited ecophenotypic plasticity and varied significantly between locations, while morphology of *Aurelia* c.f. sp. 2 was geographically invariant (i.e., canalized). The plastic, generalist medusae of *Aurelia* sp. 9 are likely able to produce environmentally-induced, “optimal” phenotypes that confer high relative fitness in different environments. In contrast, the non-plastic generalist individuals of *Aurelia* c.f. sp. 2 likely produce environmentally-independent phenotypes that provide the highest fitness across environments. These findings suggest the two *Aurelia* lineages co-occurring in the GoM were likely exposed to different past environmental conditions (i.e., different selective pressures) and evolved different strategies to cope with environmental variation. This study highlights the importance of using genetics and morphometric data to understand jellyfish ecology, evolution and systematics.

## Introduction

The degree of phenotypic differentiation in response to local environmental conditions is a crucial component to species adaptation and persistence [1,2]. Individuals in populations distributed along environmental gradients often display local phenotypes as a result of genetic adaptation and/or phenotypic plasticity [3–5], the capacity of a genotype to display a range of phenotypes in response to environmental variation [6]. Without the homogenizing effects of gene flow between selecting environments, natural selection can favor the evolution of locally adapted, genetically distinct ecotypes [7,8]. On the other hand, unrestricted gene flow can overcome phenotypic divergence via natural selection and favor the evolution of phenotypic plasticity [4,9]. Moreover, even when gene flow is high, depending on costs and limits of phenotype and plasticity [10], a geographically unstructured (i.e., environmentally canalized), generalist phenotype may evolve and be expressed throughout a species’ range [11,12].

Strategies leading to the expression of local or generalist phenotypes have been studied in several terrestrial and aquatic species [4,8,12,13], but remain unstudied in many marine planktonic groups, such as the socio-economically and ecologically important jellyfishes (i.e., medusae and ctenophores). These gelatinous animals are receiving special attention due to the capacity of a few species to suddenly increase their population size, often resulting in mass occurrences[14]. When abundant, jellyfish can considerably impact food web dynamics [15–18] and negatively affect fisheries [19–21], power and desalination plants [22,23], and tourism [24].Recent studies have linked mass occurrences of jellyfish with climatic fluctuations[25,26], overfishing, pollution, and the translocation of species [17,27]; however, the mechanisms behind mass occurrences remain poorly understood in part due to the insufficient knowledge on the ecological strategies jellyfish have evolved to cope with environmental variation [28,29].

One key step towards a better understanding of ecological strategies in jellyfish is to assess environmentally-induced effects on the phenotype of medusae in natural settings, since the phenotype is directly linked to an organism’s performance (e.g., feeding capacity, swimming ability, reproductive output) and fitness [30]. However, the extent of intraspecific, environmentally-induced phenotypic variation in jellyfish is difficult to assess mainly due to taxonomic uncertainties within this group, since most traditionally morphological features are not diagnostic at the species level [31]. Recently, the use of molecular genetics combined with explicit, quantitative, and objective morphological analyses has helped to solve prior taxonomic issues with jellyfish and uncovered cryptic speciation [31–33],as well as phenotypic variation within species [31,34,35]. Nonetheless, the extent of intraspecific phenotypic variation in jellyfish within an ecological context remains almost unexplored.

The Gulf of Mexico (GoM) represents an ideal natural setting to study phenotype-environment interactions in jellyfish populations, since several species, including the blooming moon jellyfish *Aurelia*, occur along a sharp north-to-south environmental gradient. For most of the year, the Central Northern Gulf of Mexico (CNGoM) waters are cooler than those of the Southeastern Gulf of Mexico (SEGoM) [36], which are greatly influenced by the warmer Caribbean Sea via the Loop Current. In addition, the CNGoM area is fresher and more productive than the SEGoM year round [36,37], due to freshwater and nutrient input by the Mississippi, Atchafalaya, Mobile, and Apalachicola rivers [38]. Such environmental variation may translate into different selective pressures that may promote local phenotypes via genetic adaptation or phenotypic plasticity depending on the degree of connectivity between locations [4,12,39], or generalist phenotypes depending on cost and limits of phenotype and plasticity [10,40]. Therefore, in this study, we performed molecular analyses to establish a phylogenetic background, reconstructed the evolutionary history, and inferred connectivity of *Aurelia* between the CNGoM and the SEGoM. Once genetic relationships were established, we then quantified intraspecific morphological variation between locations to specifically test for the presence of 1) a geographically invariant, generalist phenotype or 2) a geographically structured, local phenotype.

## Materials and Methods

### Medusae collection

**Fig 1:**
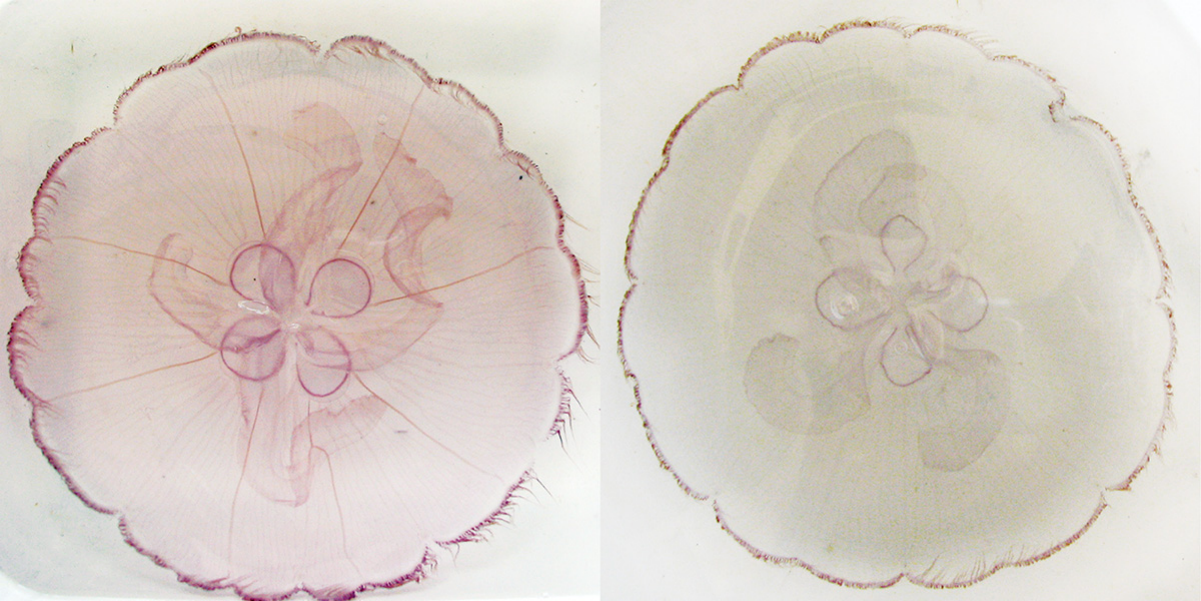
*Aurelia* medusae from the Gulf of Mexico (GoM). The specimen on the left represents a medusa of *Aurelia* sp. 9, while the specimen on the right represents a medusa of *Aurelia* c.f. sp. 2. *Aurelia* lineages in the GoM were determined by molecular analysis of mitochondrial (*COI*) and nuclear (*ITS-1*) DNA. These two particular specimens were collected in Long Key (FL) on September 28, 2006.

**Fig 2:**
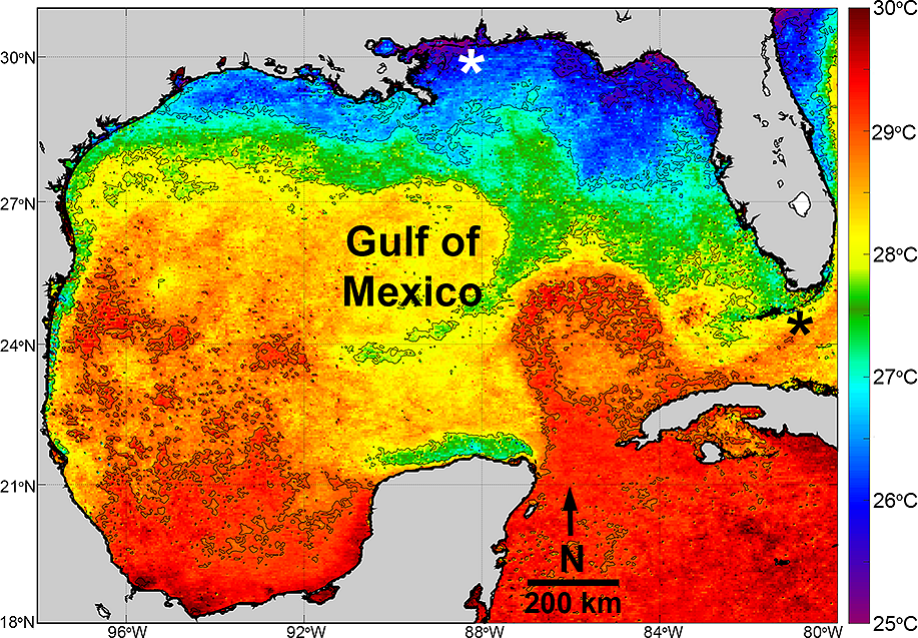
MODIS-Aqua image of sea surface temperature in the Gulf of Mexico during Fall. Sampling sites are represented by asterisks. Dauphin Island, AL (white asterisk) represents the Central Northern GoM, while Long Key, FL (black asterisk) represents the Southeastern GoM. This image is 10-day composite of sea surface temperature (4 km resolution. Level 2) from October 20 to October 30 2003. Isothermal lines were obtained using the “m_countour” function in MatLab. Image was obtained from the Ocean Color data website (http://oceandata.sci.gsfc.nasa.gov) and can be accessed at http://doi.org/10.5067/AQUA/MODIS_OC.2014.0.

*Aurelia* spp. medusae (Fig 1) were studied in the Central Northern (CNGoM: Dauphin Island, 30°09’28.6”N; 88°08’22”W) and in the Southeastern Gulf of Mexico (SEGoM: Long Key, 24°46’51”N; 80°41’12”W; Fig 2).

Individuals were sampled in the CNGoM in September and October 2003 (n = 63) and 2005 (n = 40), and in the SEGoM during September 2006 (n = 41). Medusae were collected from the surface using a dip net, placed in plastic containers with unfiltered seawater, and then transported to the laboratory. This research did not require collection permits.

### Molecular data collection

DNA was extracted from gonad tissue using a modified CTAB method that allows for multiple rounds of concentrated CTAB addition [41]. Mitochondrial cytochrome c oxidase I (*COI*) and nuclear internal transcribed spacer region 1 (*ITS-1*) were used to determine species-level variation in this study [42–44]. Polymerase chain reaction (PCR) was used to amplify *COI* using the primers LCOjf (GGTCAACAAATCATAAAGATATTGGAAC) [44] and HCO2198 (TAAACTTCAGGGTGACCAAAAAATCA) [45] and *ITS-1* region (including some flanking regions of *18S* and *5*.*8S*) was amplified using primers KMBN-8 (ATTACGTCCCTGCCCTTTGTA) and KMBN-84 (TTGCGTTCAAAGATTCGATGA) [46]. Reaction conditions for *COI* consisted of one cycle of 94°C for 180 seconds (s), then 38 cycles of 94°C for 45 s, 49°C for 60 s and 72°C for 75 s, followed by a final step of 72°C for 600 s and storage at 4°C. Reaction conditions for *ITS-1* consisted of one cycle of 94°C for 180 s, then 38 cycles of 94°C for 45 s, 50°C for 60 s and 72°C for 90 s, followed by a final step of 72°C for 600 s and storage at 4°C. PCR success was evaluating by running amplicons out on a 1.5% agarose gel. C*OI* amplicons were directly DNA-sequenced in both directions using the PCR primers, while *ITS-1* amplicons were first cloned using the TOPO TA Cloning Kit for Sequencing (Invitrogen, Inc.) and then bi-directionally sequenced using primers T3 (ATTAACCCTCACTAAAGGGA) AND T7 (TAATACGACTCACTATAGGG). Sequences were assembled using Sequencher v.5.4 (Gene Codes Corp.) and then compared to the NCBI Genbank database using BLASTx (*COI*), which translates the protein coding region and helps identify any coding issues, or BLASTn (*ITS1*). All published sequences are available in NCBI GenBank under accession numbers KU984335-KU984424.

### Molecular data analysis

Mitochondrial *COI* sequences were aligned using ClustalX v2.0 [47] and translated into amino acid to assess any coding issues. Nuclear *ITS-1* DNA sequences were aligned using MAFFT v. 7.0 employing the E-INS-I strategy [48], since it has been demonstrated to be effective for loci containing conserved regions within hypervariable regions [49]. To compare genetic divergences among *Aurelia* lineages in this study to those among animals from other studies, *COI* sequences were obtained from NCBI Genbank (S1 Table) and included in the *COI* dataset. *ITS-1* was included in this study for two reasons: to further compare genetic divergences among lineages to those among other *Aurelia* species and to look for any evidence of hybridization between GOM *Aurelia* lineages. However, initial alignments indicated that *ITS-1* regions for *Aurelia* sp. 2, *Aurelia* c.f. sp. 2 and *Aurelia* sp. 9 are distantly related to most published sequences (e.g. [42] [50]) resulting in a preponderance of poorly aligned hypervariable regions and an unreliable alignment for examining divergence among closely related species. In order to be as conservative as possible, the *ITS-1* data set for this study only included published sequences from the closely related species *Aurelia* sp. 9 (Gulf of Mexico) and *Aurelia* sp. 2 (Brazil) [42]. For *ITS-1*, hypervariable regions of questionable alignment were removed from the MAFFT alignment using GBlocks v. 0.91b [51] under default parameters, except that allowed gap positions were set to half.

Phylogenetic relationships were examined using both maximum likelihood (ML) and Bayesian inference (BI) analyses. Maximum Likelihood phylogenetic trees were constructed using PhyML 3.0 [52], employing the best-fit model chosen using jMODELTEST2 v.2.4.1 [53] using three criteria (Akaike information criterion [AIC], Bayesian information criteria[BIC] and Decision Theory Performance-Based Selection [DT].For *COI*, all criteria agreed on TrN+I+G (-lnL 4725.1989) as the best-fit model, while K80+G (-lnL 968.30284) was favored by BIC and DT for *ITS-1*. Bootstrap support was determined based on 1000 PhyML replicates. Bayesian Inference on *COI* and *ITS-1* was carried out using Mr. Bayes v.3.2.6 [54].The same model (GTR+I+G, with the gamma distribution approximated by four discrete categories) of nucleotide evolution was assumed for all analyses because it is not possible to implement the slightly less complicated models used in the ML tree searches using the COI (TRN+I+G) and ITS-1 (K80+G) data sets. For each dataset, two independent MCMC runs were conducted until the standard deviation of split frequencies decreased to less than 0.01: 2,000,000 sampling every 1,000, for the COI and ITS-1 data sets. The number of generations was determined by assessment of convergence using the Minimum Estimated Sample Size and Potential Scale Reduction Factor, as implemented in Mr. Bayes. Posterior probabilities were calculated using all trees other than the first 25%, which were discarded as “burnin”. The *COI* phylogenetic tree was rooted using a *COI* sequence from *Phacellophora camtschatica* (GQ120097), the jellyfish species most closely related to the genus *Aurelia* [55]. The *ITS-1* tree was midpoint-rooted, since the addition of distantly related *Aurelia* species introduced poorly aligned hypervariable regions, altering in-group divergences, but large-scale relationships were generally unchanged for midpoint rooted and outgroup rooted trees (not shown). All trees were constructed using Figtree v. 1.3.1 [56] and redrawn in Adobe Illustrator CS6.

Minimum inter-clade and mean intra-clade sequence divergence values and nucleotide statistics were determined using MEGA v4.0 [57] and Seaview v4.6 [58]. To assess potential genetic population structure, Analysis of Molecular Variance (AMOVA) was performed in Arlequin v.3.5.1.3[59] using *COI* sequences. A statistical parsimony haplotype network was calculated in TCS v. 1.21 [60] using the 95 per cent connection limit criterion.

### Morphological data collection

**Fig 3:**
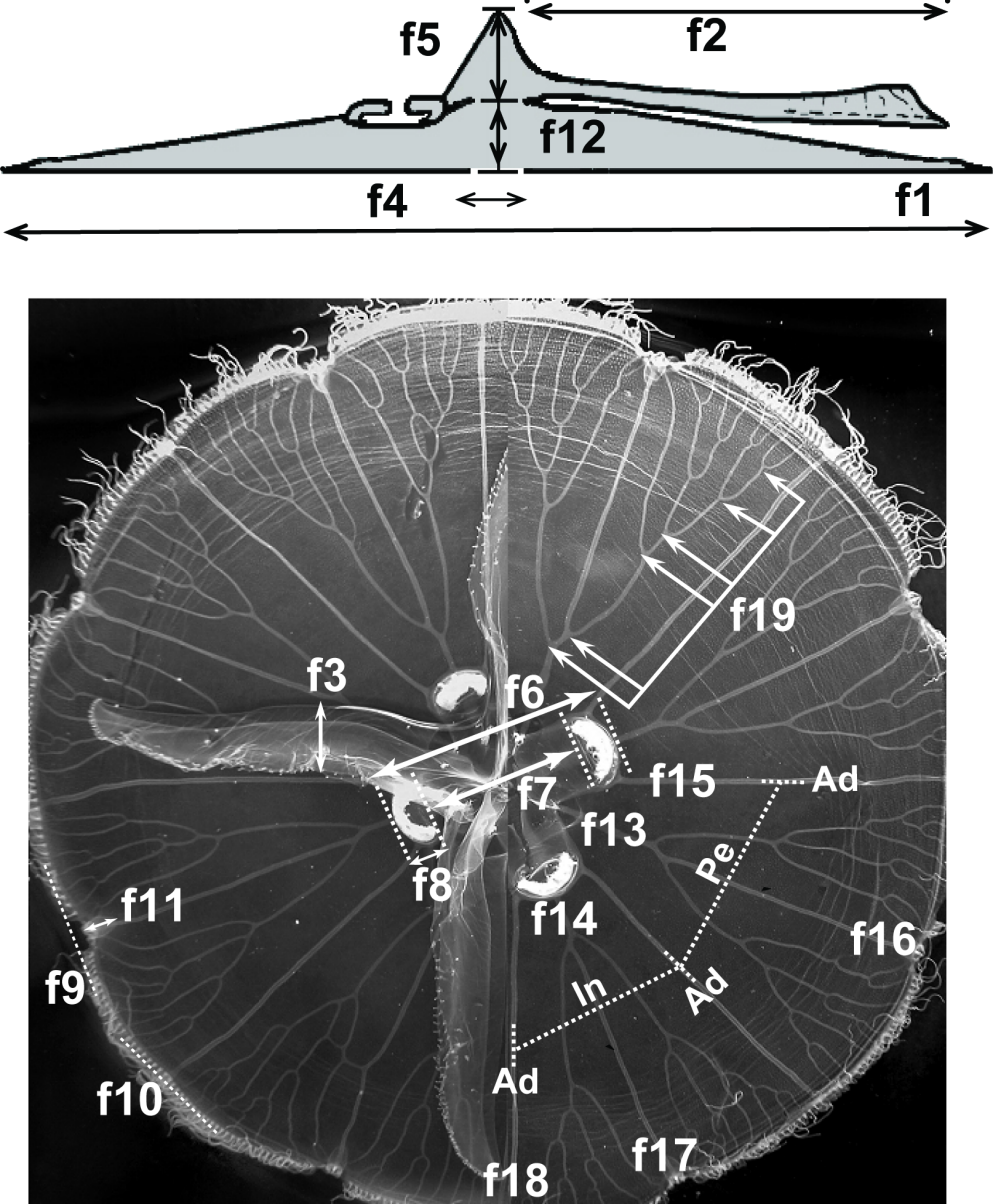
Continuous and meristic morphological features recorded in this study. Cross-sectional (A) and sub-umbrellar (B) views of an *Aurelia* medusa. Categorical features are not shown. Refer to S2 Table for feature descriptions.

A total of 28 morphological features was recorded for each *Aurelia* medusa (S2 Table). The term “morphological feature” used here refers to continuous, meristic and categorical (nominal and ordinal scales) traits previously used in morphological studies of *Aurelia* [31,61]. Features were measured and recorded based on methods described by Dawson [31], with a few modifications. Each medusa was placed with its exumbrella facing downward on a flat, transparent surface illuminated from below and digitally photographed against a 24-color Kodak reference chart and a ruler. In order to minimize handling time of individuals, only three features were recorded *in situ* from live animals. We first recorded bell diameter and then total height (bell height + manubrium length) by inserting a calibrated probe through the mouth. Oral arms and manubrium were excised and the calibrated probe was reinserted to measure bell height at the center of the bell (Fig 3). Bell height was then subtracted from total height to estimate manubrium length. The rest of the morphological features (S2 Table, Fig 3) were recorded from digital photographs using the software ImagePro Plus® v.4.5 (Media Cybernetics, Inc 1993–2001). At least two measurements per specimen were recorded for oral arm length, oral arm width, manubrium width, distal and proximate gastric distances, gonad size, rhopalium length, and rhopalar and non-rhopalar indentations (Fig 3). A series of meristic features were obtained by quadrant, including the number of perradial, interradial, and adradial originations and anastomoses (Fig 3). In addition, the maximum number of branching points of the gastrovascular system (Fig 3) was recorded per medusa according to Miyake et al. [62] and used as a proxy for relative age, since this feature increases over time in *Aurelia* medusa independently of environmental conditions [62]. Several categorical features were also recorded per medusa, including color of gastric and gonad tissue, color of umbrella, bell margin, and gastrovascular canals, shape of bell and gonads, degree of folding of the oral arms, and degree of thickening of the mesoglea surrounding the sub-genital pore (S2 Table).

### Morphological data analysis

#### Bell diameter

Differences in bell diameter versus age (number of branching points) between locations (CNGoM and SEGoM), and between years (2003 and 2005) within the CNGoM, were tested by ANOVA, and Tukey’s tests were performed for multiple comparisons.

#### Size correction approach

Since morphological features can depend on the size of the individual rather than species or population level characteristics within a particular location [31,63], all continuous, meristic, and ordinal categorical features were size-adjusted before any analysis using the method of Lleonart [64]. This procedure scales all individuals to the same size (i.e., bell diameter) and adjusts each morphological feature taking into account potential allometric differences among species or within species in different habitats using the formula:

$$Y^{*}=({{f1}_{m}/{f1}_{i})}^{b}$$ (Eq 1)

where Y* is the size-corrected morphological feature in the individual i_,_ *Y*_*i*_ is the measured morphological feature in individual i, *f1*_*i*_is the bell diameter of the individual *i*, *f1*_*m*_ is the mean (arithmetic) bell diameter of all individuals in the dataset, and *b* is the within-location, within-lineage, slope of the regression between *log*_*10*_ *(f1*_*i*_*)* and *log*_*10*_ *(Y*_*i*_*)*. Correlations were used to determine whether significant relationships existed between bell diameter and size-corrected continuous (Pearson correlations) and ordinal categorical (Spearman correlations) features using the software Statistica v.6 (Statsoft, Inc.). Nominal categorical features exhibiting a significant correlation with size were not included in subsequent analyses.

#### Multivariate analysis

Three resemblance matrices were obtained using size independent 1) continuous (Euclidean distances), 2) meristic (Bray-Curtis similarity) and 3) categorical (Bray-Curtis similarity) features in order to discern which type of features yielded better morphological differentiation. Euclidean distances are sensitive to differences of scale, thus all continuous features were first re-scaled between zero and one by dividing each observed value by the maximum value recorded for that feature. Overall morphological dissimilarity among individuals was assessed by multi-dimensional scaling (MDS) and hierarchical clustering (Cluster) analyses. Morphologically homogeneous clusters were determined by similarity profiles (SIMPROF) at *α* = 0.001. Morphological differences among clusters established by SIMPROF were quantified by means of similarity analysis (ANOSIM), and similarity percentages (SIMPER) were used to determine the relative contribution of features to morphological dissimilarity between clusters. All multivariate analyses were performed in Primer v.6 [65].

#### Univariate analysis

Differences in size-corrected continuous and meristic features were tested by two-way ANOVAs with “clade” and “location” as fixed factors. Tukey’s tests were used for multiple comparisons. Differences in ordinal categorical features were tested by a one-way approach using non-parametric *Kruskal-Wallis* and multiple pair-wise tests were performed. Differences in nominal categorical features were tested through a one-way approach using a multiple *χ*^*2*^ test for homogeneity of proportions (*Ho* = homogeneous frequency of states) and the corresponding *χ*^*2*^ pair-wise comparisons.

## Results

### Molecular analysis

#### Mitochondrial *COI*

DNA from a total of 29 *Aurelia* individuals (14 from CNGoM, 15 from SEGoM) were PCR-amplified and DNA sequenced, resulting in an aligned data set of 655 bases for sequences generated for this study. Including the 29 published *Aurelia* sequences, 231 bases of the 655-base dataset were variable, with 219 being parsimony informative. Of the 231variable sites, 42 (18.2%) occurred at first position bases, 2 (0.9%) at second position and 187 (81.0%) at third position. Base frequencies were: A = 23.8%, C = 21.3%, G = 20.4% and T = 34.6%.

**Fig 4:**
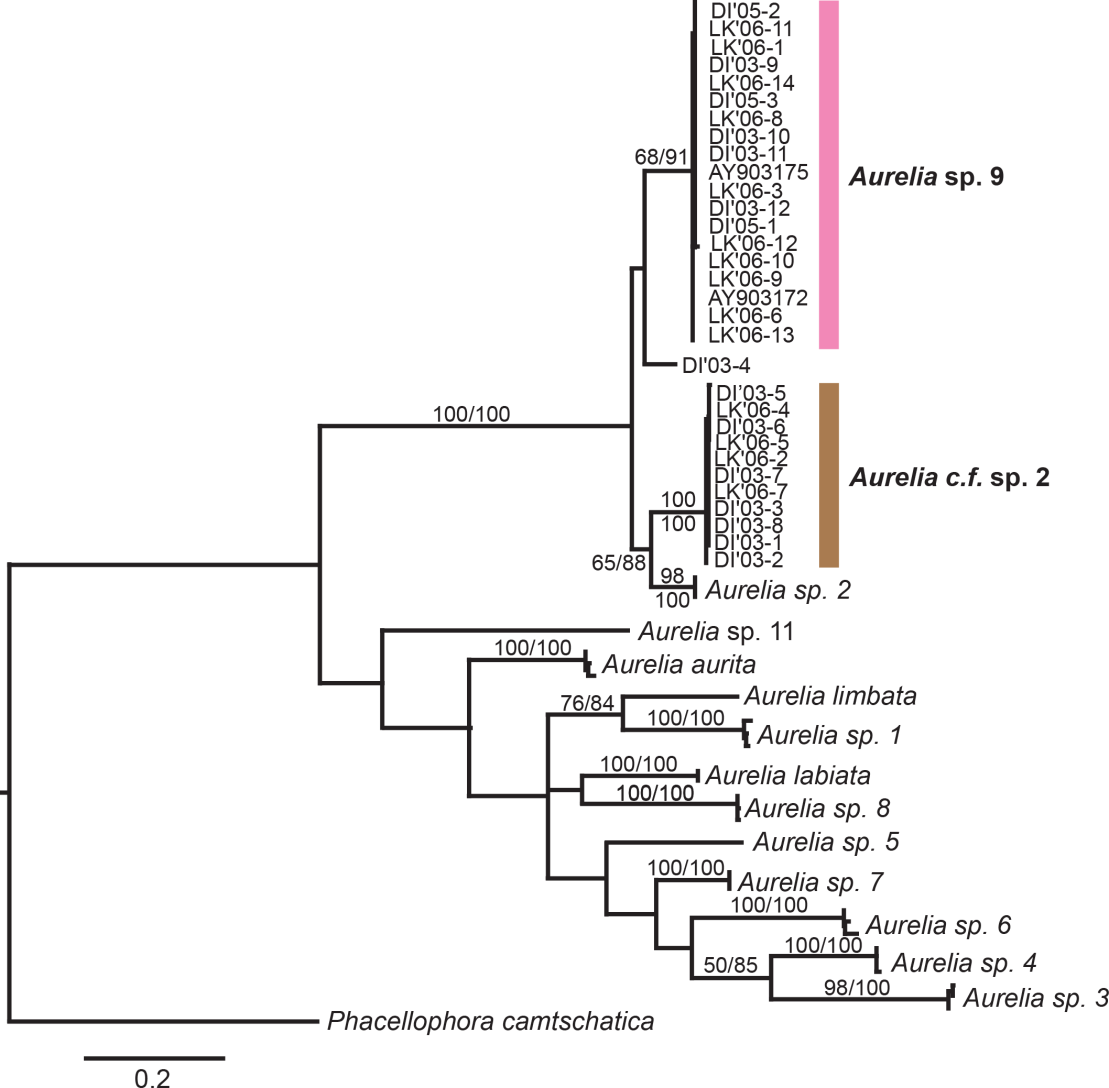
Global phylogeny of *Aurelia* based on cytochrome C oxidase subunit 1 (*COI*). Numbers indicate bootstrap values higher than 50% (maximum likelihood, 1,000 realizations). DI: Dauphin Island, Alabama. LK: Long Key, Florida. Sequences obtained in this study are shaded. The rest of sequences were obtained from GenBank (see S1 Table for details). *Aurelia aurita* (Turkey, Sweden, Boston-USA), 2) *Aurelia labiata* (British Columbia-Canada), 3) *Aurelia* sp. 1 (Los Angeles-USA, Australia, Japan), 4) *Aurelia* sp. 2 (Brazil), 5) *Aurelia* sp. 3 (Palau), 6) *Aurelia* sp. 4 (Hawaii-USA, Indonesia, Palau), 7) *Aurelia* sp. 5 (Mljet Lake, Croatia), 8) *Aurelia* sp. 6 (Palau, New Guinea), 9) *Aurelia* sp. 7 (Tasmania, Australia), 10) *Aurelia* sp. 8 (North Adriatic, Croatia), 11) *Aurelia* sp. 9 (Gulf of Mexico, USA; this study), 12) *Aurelia limbata* (*Aurelia* sp. 10, Japan), 13) *Aurelia* sp. 11 (Marshall Islands), 14) *Aurelia* c.f. sp. 2 (Gulf of Mexico, USA; this study), 15) *Aurelia* sp. DI’03–4 (Gulf of Mexico, USA; this study).

The *COI* maximum likelihood phylogeny indicated that most of the *Aurelia* sequences generated for this study fell into two distantly related (minimum: 11.8% divergence; average: 12.7% divergence; S3 Table), well-supported monophyletic groups (Fig 4), with 17 specimens forming a clade with *Aurelia* sp. 9 from the Gulf of Mexico [44] and 11 individuals forming a distinct clade most closely related (minimum 11% divergence, average 11.5%; S3 Table) to *Aurelia* sp. 2 from Brazil [42] (Fig 4). This clade, referred to here as *Aurelia* c.f. sp. 2 (see Discussion), is reciprocally monophyletic to both *Aurelia* sp. 2 and *Aurelia* sp. 9 (Fig 4). In addition, one individual (DI’03–4) did not group with any species, differing from *Aurelia* sp. 9, *Aurelia* c.f. sp. 2, and *Aurelia* sp. 2 by 9.8%, 12.7%, and 11.4% (minimum sequence divergence), respectively. However, since this specimen did not correspond to a well-represented monophyletic clade, its phylogenetic status will not be discussed further.

Statistical parsimony network analysis of *COI* sequences showed that the most common haplotypes of each *Aurelia* lineages were shared by individuals from both the CNGoM and the SEGoM (S1 Fig). Haplotype diversity for *Aurelia* sp. 9 was– 0.86 ± 0.14 in the CNGoM and 0.84 ± 0.10 in the SEGoM. Haplotype diversity for *Aurelia* c.f. sp. 2 was 1.0 ± 0.08 in the CNGoM and 1.0 ± 0.18 in the SEGoM. AMOVA detected no significant geographic population sub-division in either *Aurelia* lineage (S4 Table), with only 6.47% (*Aurelia* sp. 9) and -1.95% (*Aurelia* c.f. sp. 2) of the total intraspecific genetic variation explained by genetic differences between locations.

#### Nuclear *ITS-1*

A total of 61 *ITS-1* clones were sequenced from 31 *Aurelia* specimens (18 from CNGoM and 13 from SEGoM) and amplified fragments (including flanking 18S and 5.8S sequence) varied in length from 652 (Clade I) to 712 (Clade II) base pairs, with all length variation (e.g. insertion/deletions) within the *ITS-1* region. After sequence alignment and the application of GBlocks to remove regions of questionable alignment, the final *ITS-1* dataset was 359 base pairs. Of the 359 base pairs, 54 were gapped, 55 were variable, non-gapped and non-degenerate, with 47 being parsimony informative. Base frequencies were: A = 20.6%, C = 26.8%, G = 29.9%, T = 22.8%.

**Fig 5:**
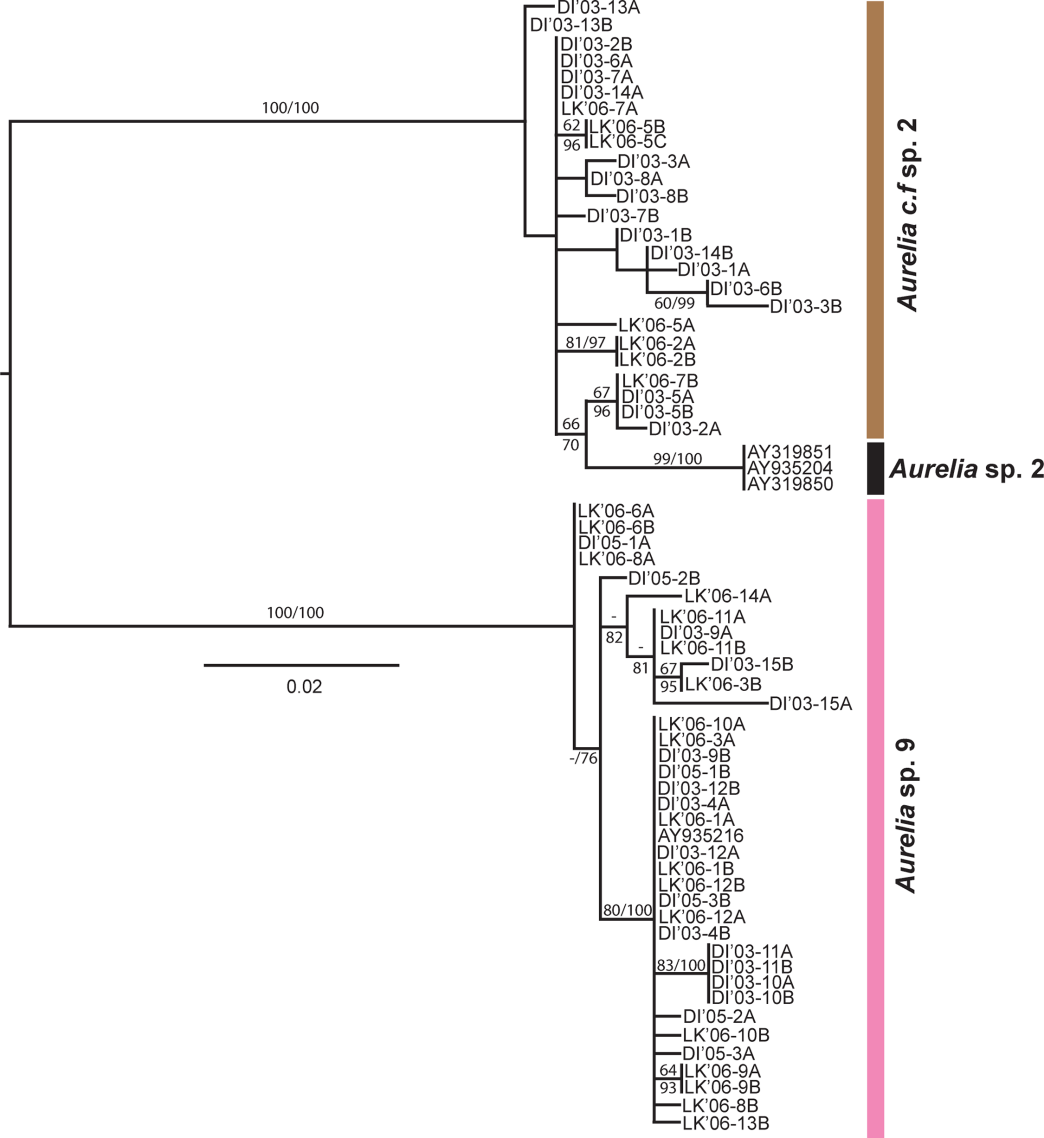
Phylogeny of *Aurelia* in the Gulf of Mexico based on *ITS-1*. Note *Aurelia* c.f. sp. 2 (based on mitochondrial *COI*) is grouped within the *Aurelia* sp. 2 clade. The tree was mid-rooted. Bootstrap values higher than 50% are shown (maximum likelihood, 1,000 realizations). Sequences obtained in this study are DI (Dauphin Island, AL) and LK (Long Key, FL). The other sequences were obtained from GenBank (see S1 Table).

**Fig 6:**
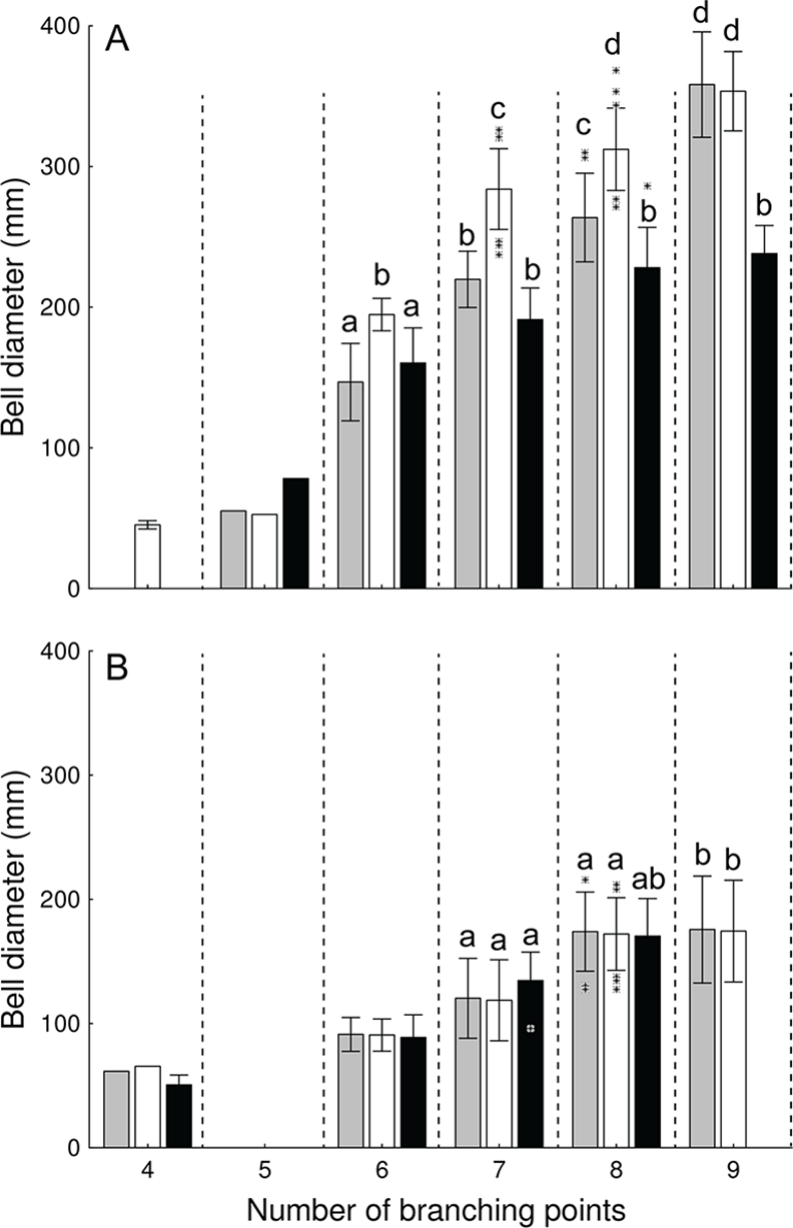
Relationship between bell diameter and number of branching points (i.e., age) in *Aurelia* medusae from the Gulf of Mexico. A: *Aurelia* sp. 9. B: *Aurelia* c.f. sp. 2. Grey (2003) and hollow (2005) bars represent medusae collected from the Central Northern Gulf of Mexico (Dauphin Island, AL), while black bars represent individuals collected in the Southeastern Gulf of Mexico (Long Key, FL 2006). Means with different letters are significantly different (Tukey’s, P< 0.001). Bars: SD. Asterisks: outliers

The smaller scale *ITS-1* phylogeny (Fig 5) was generally similar to that found for *COI*, but with lowered clade divergence in some cases. Thirty-six clones from19 individuals formed a clade with *Aurelia* sp. 9 [44], while 25 clones from 12 individuals formed a clade similar to *Aurelia* sp. 2 from Brazil [42], with a minimum genetic divergence of 9.2% between clades (Fig 6). The animals from the second, *Aurelia* sp. 2-like (*Aurelia* c.f. sp. 2 based on *COI*) clade, still differed from *Aurelia* sp. 2 by a minimum sequence divergence of 1.9% (Average: 2.3%), but did not form a separate, well-supported monophyletic group (Fig 5).

### Morphological analysis

#### Bell diameter

Since molecular analyses revealed the presence of two distantly related *Aurelia* monophyletic groups based on both *COI* and *ITS-1*, variation in bell diameter with age was analyzed for each clade separately. Medusae of each clade were split into three groups: I) NGoM (2003), II) NGoM (2005), III) SEGoM (2006). Differences in bell diameter versus age, between locations and years, were then tested with two-way ANOVAs, with “group” and “age” (number of branching points) as fixed factors. ANOVA detected a significant “group x age” effect on bell diameter of *Aurelia* sp. 9 (F_6,60_ = 49, P< 0.001) Medusae of *Aurelia* sp. 9 from the CNGoM collected in 2003 had significantly larger bell diameters at 6, 7, and 8 branching points than their counterparts from the same location collected in 2005 (Tukey’s, P< 0.05), but they reached a similar size at 9 branching points (Fig 6A). *Aurelia* sp. 9 medusae from the CNGoM collected in 2003 and 2005 had significantly larger bell diameters at any given number of branching points and at eight and nine branching points, respectively, than their counterparts from the SEGoM (Tukey’s, P< 0.05; Fig 6A). In contrast, ANOVA detected only a significant “age” affect on the bell diameter of *Aurelia* c.f. sp. 2 (F_6,49_ = 49, P< 0.01), indicating that bell diameter did not differ between locations, or years, at any given branching point (ANOVA,P> 0.05; Fig 6B).

#### Continuous features

**Fig 7:**
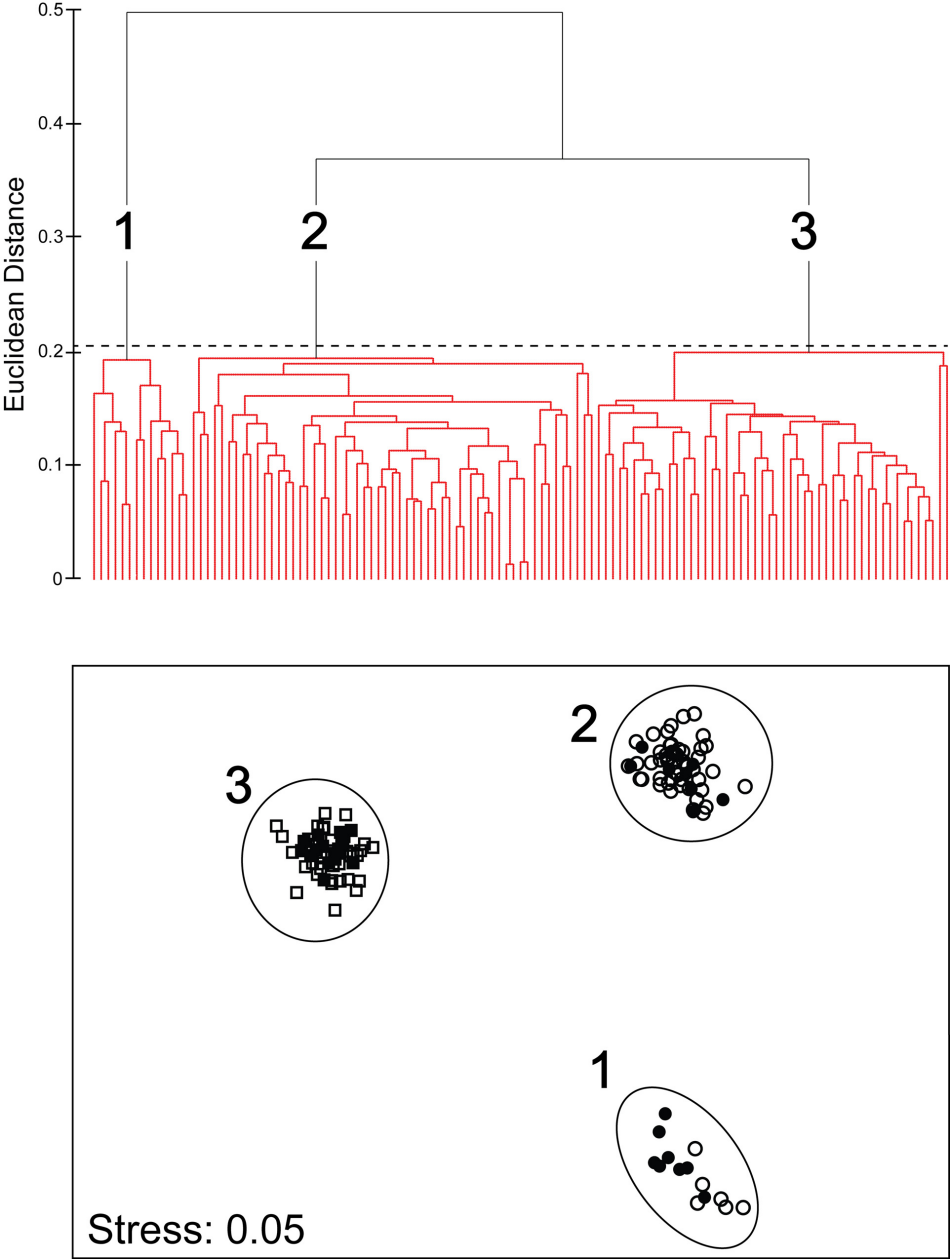
Morphological variation in *Aurelia* medusae from the Gulf of Mexico based on continuous (size-corrected) morphological features. Top: Cluster analysis. Red lines indicated morphologically homogeneous clusters detected by SIMPROF. Bottom: Multi-dimensional scaling analysis (MDS). Different symbols indicate different monophyletic *Aurelia* clades based on *COI*. Circles: *Aurelia* sp. 9. Squares: *Aurelia* c.f. sp. 2. Solid symbols represent individuals with molecular + morphological data, while open symbols are individuals with only morphological data. Cluster 1: *Aurelia* sp. 9 from Long Key (2006). Cluster 2: *Aurelia* sp. 9 from Dauphin Island (2003 and 2005). Cluster 3: *Aurelia* c.f. sp. 2 from Dauphin Island (2003 and 2005) and Long Key (2006). Contours represent 95% confidence.

Hierarchical Cluster Analysis (Cluster) and Multi Dimensional Scaling (MDS) analyses grouped all *Aurelia* medusae into three morphologically homogeneous, non-overlapping clusters (SIMPROF: π = 0.83, P< 0.005; Fig 7), which included all *Aurelia* sp. 9 medusae collected from the SEGoM (Cluster 1), all *Aurelia* sp. 9 individuals collected from the CNGoM (Cluster 2) and all *Aurelia* c.f. sp. 2 specimens collected from both the CNGoM the SEGoM (Cluster 3), respectively (Fig 7). ANOSIM revealed a significant morphological dissimilarity among all clusters (*R* = 0.86, P<0.0001, all pair-wise comparisons).

**Fig 8:**
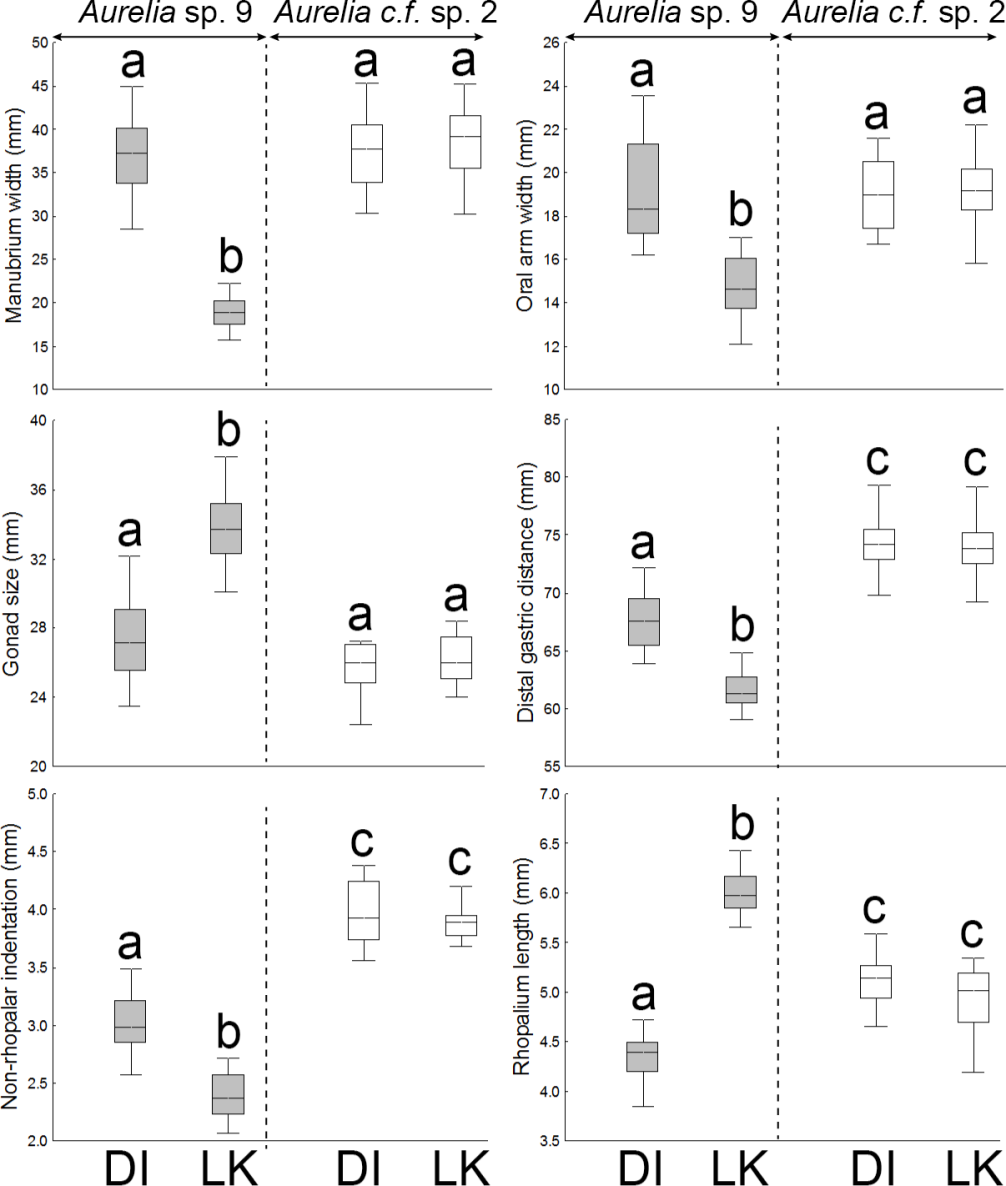
Morphological variation in continuous, size-corrected features of *Aurelia* medusae in the Gulf of Mexico. DI: Dauphin Island (AL), which represents the Central Northern Gulf of Mexico. LK: Long Key (FL), which represents the Southeastern Gulf of Mexico. Features explaining 90% of the total morphological variation (SIMPER) are shown. Different letters above whiskers indicate significant differences among groups detected by post-hoc Tukey’s tests (P < 0.0001). Boxes: median, the 25–75% quartiles. Whiskers: Non-outlier range. Circles: outliers

None of the 11 continuous features recorded per medusae varied significantly between locations in *Aurelia* c.f. sp. 2; however, 10 features of *Aurelia* sp. 9 displayed significant geographic structure (S5 Table). SIMPER indicated that most (90%) geographic morphological variation in *Aurelia* sp. 9 was due to variation in six features, including manubrium width (30.6%), oral arm width (17.4%), gonad size (13.2%), distal gastric distance (10.3%), non-rhopalar indentation (10.1%), and rhopalium length (8.4%). *Aurelia* sp. 9 medusae from the NGoM had significantly wider and longer manubria, wider and longer oral arms, longer proximal and distal gastric distances, smaller rhopalia, smaller gonads, thinner umbrellas, and deeper non-rhopalar indentations, than individuals from the SEGoM (Tukey’s, P< 0.01; Fig 8).

Six features varied between *Aurelia* sp. 9 and *Aurelia* c.f. sp. 2 independently of location, including manubrium length, oral arm length, distal and proximal gastric distance, depth of non-rhopalar indentations, and bell thickness (Tukey’s, P < 0.01; Fig 8). In addition, four features varied significantly between clades, but were location-dependent. These included manubrium and oral arm width, length of rhopalium, and gonad size (Tukey’s, P < 0.01; Fig 8).

#### Categorical features

**Fig 9:**
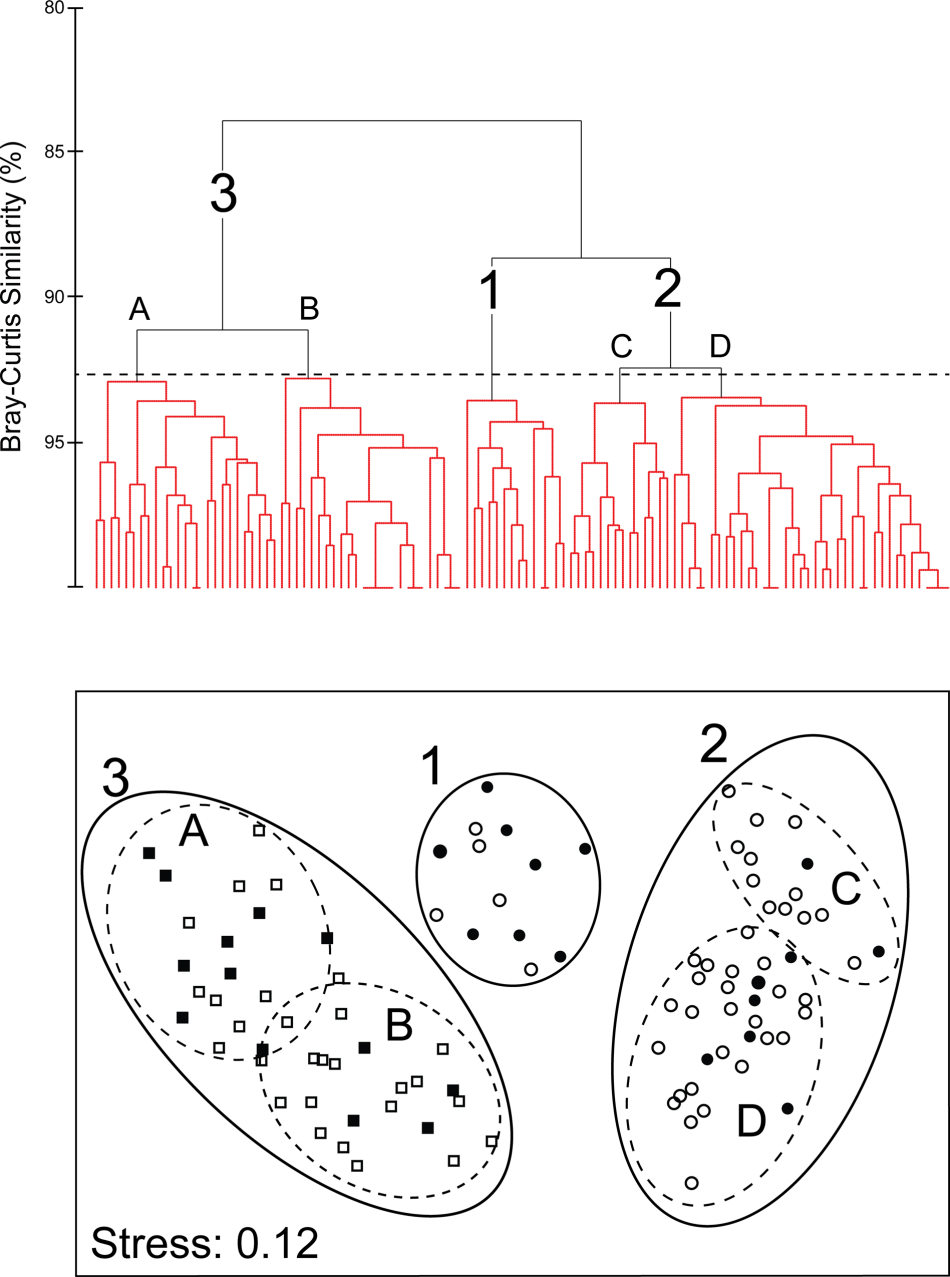
Morphological variation in *Aurelia* medusae from the Gulf of Mexico based on categorical morphological features. Top: Cluster analysis. Red lines indicated morphologically homogeneous clusters detected by SIMPROF. Bottom: Multi-dimensional scaling analysis (MDS). Numbers correspond to the same main clusters detected by SIMPROF using continuous features. Letters indicate sub-clusters within main clusters. Different symbols indicate different monophyletic *Aurelia* clades based on COI. Circles: *Aurelia* sp. 9. Squares: *Aureli*a c.f. sp. 2. Solid symbols represent individuals with molecular + morphological data, while open symbols are individuals with only morphological data. Cluster 1: *Aurelia* sp. 9 from Long Key (2006). Cluster 2: *Aurelia* sp. 9 from Dauphin Island (2003 and 2005). Cluster 3: *Aurelia* c.f. sp. 2 from Dauphin Island (2003 and 2005) and Long Key (2006). Contours represent 95% confidence.

Cluster and MDS analyses grouped individuals in the same three main clusters obtained using continuous features (Fig 9). SIMPROF analysis, however, detected morphological subdivision within Cluster 3 (*Aurelia* c.f. sp. 2, Sub-clusters A and B) and Cluster 2 (*Aurelia* sp. 9 from the CNGoM, Sub-clusters C and D; π = 2.1, P< 0.002; Fig 9). ANOSIM indicated significant morphological dissimilarity among all clusters detected by SIMPROF(R = 0.89,P< 0.001, p < 0.001 all pair wise comparisons). A certain degree of geographic sub-division was displayed by *Aurelia* c.f. sp. 2 medusae, with the majority (68%) of individuals collected from the CNGoM forming sub-cluster A, and most individuals from the SEGoM (87%) being included in sub-cluster B.

**Fig 10:**
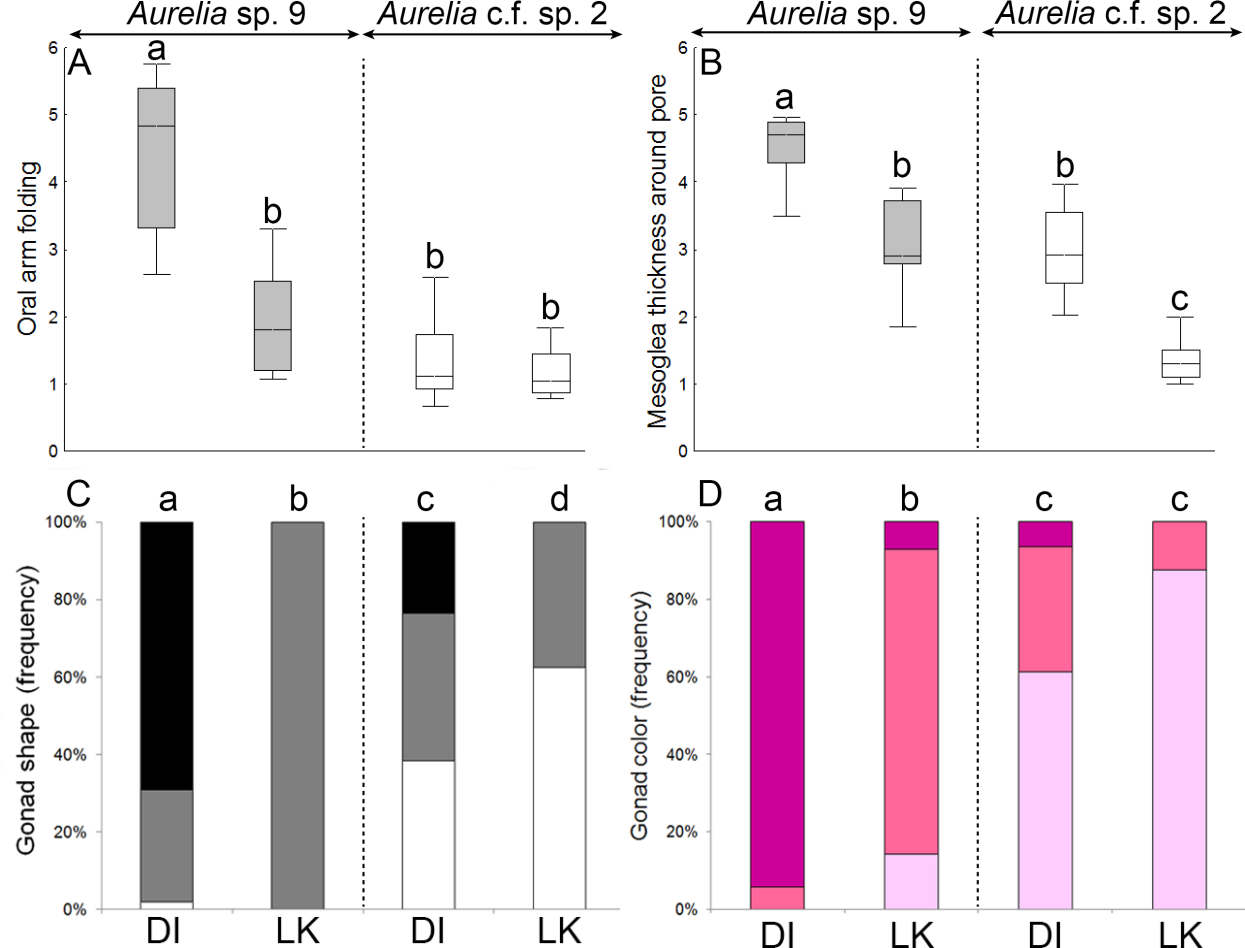
Morphological variation in categorical features of *Aurelia* medusae in the Gulf of Mexico. A-B: Ordinal categorical features. C-D: nominal categorical features. DI: Dauphin Island (AL), which represents the Central Northern Gulf of Mexico. LK: Long Key (FL), which represents the Southeastern Gulf of Mexico. Features explaining 90% of the total morphological variation (SIMPER) are shown. Different letters above whiskers indicate significant differences among groups detected by post-hoc Kruskal-Wallis tests (P< 0.001; A-B) and *Chi-square* tests (P < 0.001; C-D). Gonad shape in panel C are: U-shape (hollow bars), horseshoe (gray bars) and drop-like (black bars). Boxes: median, the 25–75% quartiles. Whiskers: Non-outlier range.

Medusae of *Aurelia* sp. 9 exhibited significant geographic variation in five out of nine categorical features recorded (S5 Table); however, SIMPER indicated the geographic variation (90%) was explained by variation in three features, including the degree of oral arm folding (51%), gonad color (24%), and gonad shape (15%). *Aurelia* sp. 9 medusae from the CNGoM had a significantly higher degree of oral arm folding (Post-Kruskal-Wallis, P< 0.001), and darker, more predominantly “drop-like” shape (*χ*^*2*^-tests, *p* < 0.001) gonads, than counterparts in the SEGoM (Fig 10).

Only two features of *Aurelia* c.f. sp. 2 varied significantly between locations (S5 Table), and each one explained 50% of the geographic morphological variation in this lineage (SIMPER). Individuals of *Aurelia* c.f. sp. 2 from the CNGoM had thicker mesoglea surrounding the sub-genital pore, and more variation in gonad shape, than individuals from the SEGoM (Fig 10).Variation in all the aforementioned features accounted for at least 90% of the total geographic morphological variation within both clades.

Four categorical morphological features differed between *Aurelia* sp. 9 and *Aurelia* c.f. sp. 2 independently of location, which included color of bell and bell margin, and shape and color of gonad tissue (Pair-wise *χ*^*2*^-tests, P< 0.001 for all features; Fig 10). Three morphological features varied significantly between clades, but were location-dependent. These included thickness of mesoglea around the sub-genital pore, degree of oral arm folding (Post-Kruskal-Wallis, P< 0.05), and color of gastric tissue (Pair-wise *χ*^*2*^-tests; *p <* 0.001; Fig 10).

#### Meristic features

Cluster and MDS analyses using meristic features did not reveal intra- or inter-specific morphological differentiation (S2 Fig). Instead, individuals were grouped in two morphologically homogeneous main clusters (SIMPROF: π = 0.15, P< 0.001) due to random variation in the number of interradial originations (90%, SIMPER), independently of clades, location, or year of collection (S2 Fig).

## Discussion

The use of molecular and morphological analysis in this study revealed the presence of two molecularly and morphologically distinct species of *Aurelia* co-occurring in the Gulf of Mexico (GoM): *Aurelia* sp. 9 and *Aurelia* c.f. sp. 2. Neither species exhibited significant population sub-division between the Central Northern GoM and the Southeastern GoM, but the degree of geographic morphological differentiation varied greatly between lineages, in spite of their co-occurrence at the extremes of a latitudinal environmental gradient. The morphology of *Aurelia* sp. 9 was clearly geographically structured (i.e., local phenotype), while the morphology of *Aurelia* c.f. sp. 2 was geographically invariant (i.e., generalist phenotype). The current study represents, to our knowledge, the first case of different ecological strategies in response to environmental variation in two sympatric, congeneric jellyfish lineages. These findings not only suggest evolution under different environmental conditions (i.e., different selective pressures) that led to the implementation of different strategies to cope with environmental heterogeneity, but also emphasize the importance of using genetics and morphometrics in concert to understand jellyfish ecology, evolution and taxonomy.

Mitochondrial (*COI*) and nuclear (*ITS-1*) DNA sequence data indicated that there are two different phylogenetic species co-occurring in the GoM. The minimum percentage sequence divergence in *COI* detected between *Aurelia* sp. 9 and *Aurelia* c.f. sp. 2 (11.8%; Average 12.7%) not only fell within the 10–20% (average) range previously considered diagnostic for delineating species of *Aurelia* [42,44], *Cassiopea* [32], *Cyanea* [66,67], and *Drymonema* [33], but also was higher than the average congeneric interspecific distance threshold (~11%) previously determined for the Class Scyphozoa [68]. In addition, minimum sequence divergence for *ITS-1* between the two GoM lineages (9.2%; average 12.7%) was also within range of average *ITS-1* sequence divergence among congeneric species of *Aurelia* [42,50,69] and *Cyanea* [66]. It should be noted that *ITS-1* divergence values are likely affected by how gapped and unalignable regions are treated and, therefore, published values are likely affected by the fact that these regions were not handled in a consistent manner across past publications (e.g.,[42,50,69]). Although individuals from the GoM (*Aurelia* c.f. sp. 2) and Brazil (*Aurelia* sp. 2) form two distantly related clades based on *COI* (minimum divergence 11.0%; average: 11.5%), they did not differ greatly for *ITS-1* (minimum: 1.9%; average: 2.3%). Hence, given these discrepancies and the fact that naming a new species is beyond the scope of this paper, we have taken the conservative approach of giving the name *Aurelia* c.f. sp. 2 to this GoM clade, in order to reflect its divergence from (based on *COI*) and similarity to (based on *ITS-1*) *Aurelia* sp. 2. However, clarifying *Aurelia* taxonomy in the western Atlantic will require additional research.

Genetic data suggests that, despite co-occurring spatially and temporally in the GoM, *Aurelia* sp. 9 and *Aurelia* c.f. sp. 2 have distinct evolutionary histories. Reconstructed phylogeny based on *COI* revealed a well supported sister-species relationship between *Aurelia* c.f. sp. 2 and *Aurelia* sp. 2 from Brazil (~7000 km south of the GoM; [42]), suggesting these two lineages likely share a recent common ancestor that is different from that of *Aurelia* sp. 9. Further support is added by the minor segregation between *Aurelia* c.f. sp. 2 and *Aurelia* sp. 2 based on *ITS-1*. Considering 0.5–1.4% of sequence divergence in *COI* per million years in several marine invertebrates [70, 71,72] including hydrozoan jellyfish [73], divergence between *Aurelia* sp. 9 and *Aurelia* c.f. sp. 2 may have occurred during the Miocene (23–7.1 Myr BP), and the lack of evidence for hybridization from *ITS-1* data suggests these species have remained reproductively isolated ever since. Estimations of divergence, however, must be taken with caution, because 1) mutation rates can be both location and taxa-specific [73], 2) reliable clock calibrations for scyphozoans have not been developed yet [74], and 3) potential saturation of mutations at neutral nucleotide positions may result in underestimations of divergence time [42].

The geographically structured morphology displayed by individuals *Aurelia* sp. 9 despite a lack of genetic structure is likely due to ecophenotypic plasticity. Several theoretical and empirical studies suggest that the homogenizing effects of high gene flow between selective environments (in this case, the CNGoM and the SEGoM) can reduce local, genetic adaptation and favor the evolution phenotypic plasticity [4,9,75], but see [76]. Gene flow can introduce genes adapted to environmental conditions that may differ from local conditions, which can be potentially maladaptive in the new environment. Therefore, phenotypic plasticity allows organisms adapt quickly to an alternate environment [75] and potentially optimize fitness [77] by shifting the phenotype of a population toward a local optimum without any genetic differentiation [78]. Thus, given the lack of significant genetic structure between sampled locations found in this study, the geographic morphological variation observed in *Aurelia* sp. 9 likely represents a plastic response to differences in local environmental conditions. In addition, models for the evolution of phenotypic plasticity indicate that the consistency of environmental cues, and the capacity of organisms to sense the environment, is of the outmost importance for plasticity to be favored within and across generations [79]. Hence, it is plausible that the ecophenotypic plasticity displayed by *Aurelia* sp. 9 is an adaptation as a result of evolution under reliable and consistent environmental variation.

Ecophenotypic plasticity in response to consistent latitudinal variation in temperature and food availability in the GoM may explain the observed geographic morphological structure displayed by *Aurelia* sp. 9. Individuals in the CNGoM had significantly larger bell diameters at same relative age than counterparts from the SEGoM. During the period of occurrence of *Aurelia* sp.9 medusae in the GoM from August to December [80], individuals from the SEGoM are exposed to 2–9 °C warmer and considerably less productive waters than their counterparts in the CNGoM [36,37]. Body size (i.e., bell diameter) has previously been shown to be negatively affected by increased temperature [81] and positively affected by increased food availability [82]. The Temperature-size Rule (TSR) [83] states that in ectotherms, increased temperatures speed up development and shorten time to reproduction, which results in early full development (and subsequently maturation) at relatively small sizes [84]. In addition, Life History Theory (LHT) [85,86] states that when food is scarce somatic growth rates slow down and energy allocation is shifted toward reproduction resulting in maturation at relatively small sizes; however, when food is abundant most energy is allocated into somatic growth at expense of gonad development and maturation is then reached at a relatively large size. The results of this study are in line with both TSR and LHT. Individuals of *Aurelia* sp. 9 from the warmer, less productive SEGoM reached maximum bell diameter at a relative younger age (lower number of branching points) than medusae from the cooler, more productive CNGoM. Similar results were observed in *Aurelia aurita* from Southampton Water, which reached full development at smaller sizes with increased temperature [81]. In addition, an increase in food supply can slow gonad development in *Aurelia* [82]; thereby, well-fed individuals reached maturity later, and at larger sizes, than food-limited medusae.

The geographic morphological variation displayed by medusae of *Aurelia* sp. 9 may translate to geographic differences in fecundity and reproductive capacity. Previous studies have shown that size is correlated with fecundity and reproductive output in jellyfish [87,88]; thereby, it is likely that *Aurelia* sp. 9 medusae from the CNGoM produce more eggs and planula larvae than individuals in the SEGoM. In *Aurelia*, female medusae are brooders, and their planula larvae are carried on the oral arms[89]. Interestingly, *Aurelia* sp. 9 medusae from CNGoM had relatively wider/longer oral arms than their counterparts from the SEGoM. It is plausible that the larger, potentially more fecund individuals from the CNGoM require a relatively larger surface to carry their planulae, which can be achieved by developing proportionally larger oral arms. In addition, medusae of *Aurelia* sp. 9 from the CNGoM had a significantly higher degree of folding of the oral arms than counterparts from the SEGoM, which increases oral arm surface even further and can potentially allow medusae to maximize their carrying capacity for planula larvae. It is curious that the degree of folding of oral arms was the only morphological feature that showed sexual dimorphism. Females had more convoluted oral arms than males, a pattern previously observed in other *Aurelia* species [31].However, sexual differences in the degree of folding of the oral arms of *Aurelia* sp. 9 were only significant in the CNGoM (data not shown). These findings suggest that the reproductive capacity of *Aurelia* sp.9 medusae is plastic. Hence, individuals from the CNGoM likely have a higher reproductive capacity than counterparts in the SEGoM, which may potentially translate into differences in larva recruitment and, subsequently medusa numbers, between these two areas. This hypothesis is supported by a previous study by Rodden et al [80], which shows that abundance of *Aurelia* medusae in the CNGoM was considerably higher than in the SEGoM.

On the other hand, considering high genetic connectivity between sampling locations in *Aurelia* c.f. sp. 2, the generalized phenotypes displayed by medusae of this species are likely the result of environmental canalization, the reduced sensitivity (or increased robustness) of phenotypes to environmental change [12]. Individuals of *Aurelia* c.f. sp. 2 exhibited no significant geographic morphological structure despite being exposed to geographic environmental variation. Environmental canalization is thought to have evolved as a product of stabilizing selection in environments with relatively low spatio-temporal variability [12]. Under stabilizing selection, a reduced environmental variation increases mean fitness; thereby, the evolution of environmental canalization is favored [11,90]. Therefore, the results of this study suggests that *Aurelia* c.f. sp. 2 may have originated in a relatively stable environment that may have favored the evolution of invariant phenotypes, likely via genetic assimilation [91], allowing *Aurelia* c.f. sp. 2’s genotype can express a non-plastic morphology despite being exposed to environmental change.

Previous studies have shown that canalizing mechanisms can be located at any level of biological hierarchy; thereby, lack of variation may be observed at the morphological level but not at the level of gene expression or physiology [92]. In addition, canalization implies a trade-off where stability at one level may depend upon variability at another level [93]. Hence, it is plausible that morphology of *Aurelia* c.f. sp. 2 is maintained invariant, despite environmental change, at the expense of non-random latitudinal variation in gene expression not detected by the methods used in this study. This phenomenon termed counter-gradient variation [94] has been previously proposed to explain phenotypic similarities among *Aurelia* species distributed along latitudinal environmental gradient [95]. Further research will be needed to elucidate the mechanisms behind the observed pattern.

Phenotypic differences between medusae of *Aurelia* sp. 9 and individuals of *Aurelia* c.f. sp. 2 suggest a direct correlation between morphological and molecular evolution. Previous studies in *Aurelia* and other scyphozoan medusae, including *Cyanea* and *Drymonema*, have also revealed congruent patterns of genetic and phenotypic divergence [31,33,35], but see [32]. Morphological variation among congeneric species often indicates reproductive isolation and evolution under different ecological conditions [96]. Therefore, based on the results of this study, we hypothesize that *Aurelia* sp. 9 and *Aurelia* c.f. sp. 2 have different evolutionary histories, and that these species evolved independently under different selective pressures (i.e, different environmental conditions) that resulted in different ecological strategies to cope with environmental variation. A similar mechanism has been proposed by Schroth et al. [50], who suggested that ecological differences observed among *Aurelia* lineages may be the result of divergent selection, with environmental factors playing a deterministic role in diversification. The plastic, generalist strategy exhibited by medusae of *Aurelia* sp. 9 in the GoM may translate in the potential expression of “optimal”, environmentally-dependent phenotypes that confer high relative fitness in different environments, while the non-plastic generalist individuals of *Aurelia* c.f. sp.2 may produce an environmentally-independent phenotype that gives the highest mean fitness across environments [10,40]. Assessing the extent and patterns of environmentally-induced intra and inter-specific phenotypic differentiation in jellyfish is necessary to comprehend mechanisms of bloom formation and to predict future mass occurrences. Considering the world’s changing ocean conditions (e.g., warming temperatures, eutrophication, acidification, etc.), it is essential to determine how this environmental change can induce phenotypic changes and the potential consequences of those changes on reproduction and fitness of jellyfish.

## Supporting Information

S1 Fig Network tree based on *COI* for *Aurelia* sp. 9 and *Aurelia* c.f. sp. 2 in the Gulf of Mexico. (DOCX)

S2 Fig Morphological variation in *Aurelia* from the Gulf of Mexico based on meristic features. (DOCX)

S1 Table List of sequences obtained from Genbank used in this study. (DOCX)

S2 Table Morphological features recorded per *Aurelia* medusa in this study. (DOCX)

S3 Table Pairwise genetic divergence among *Aurelia* species based on *COI*. (DOCX)

S4 Table Summary of the AMOVA based on COI for *Aurelia* sp.9 and *Aurelia* sp.12 in the Gulf of Mexico. (DOCX)

S5 Table Variation in individual size-independent morphological features of *Aurelia* from the Gulf of Mexico. (DOCX)
