# Correction: Local versus Generalized Phenotypes in Two Sympatric *Aurelia* Species: Understanding Jellyfish Ecology Using Genetics and Morphometrics

**DOI:** 10.1371/journal.pone.0162118

**Published:** 2016-08-25

**Authors:** Luciano M. Chiaverano, Keith M. Bayha, William M. Graham

The second author's name is spelled incorrectly. The correct name is: Keith M. Bayha. The correct citation is: Chiaverano LM, Bayha KM, Graham WM (2016) Local versus Generalized Phenotypes in Two Sympatric *Aurelia* Species: Understanding Jellyfish Ecology Using Genetics and Morphometrics. PLoS ONE 11(6): e0156588. doi:10.1371/journal.pone.0156588
