# Supplementary material for: Local versus Generalized Phenotypes in Two Sympatric Aurelia Species: Understanding Jellyfish Ecology Using Genetics and Morphometrics
Source: PLoS One. 2016 Jun 22;11(6):e0156588. doi: 10.1371/journal.pone.0156588 (PMC4917110; doi:10.1371/journal.pone.0156588)
Supplement: S1 Fig — (DOCX) [file pone.0156588.s001.docx]

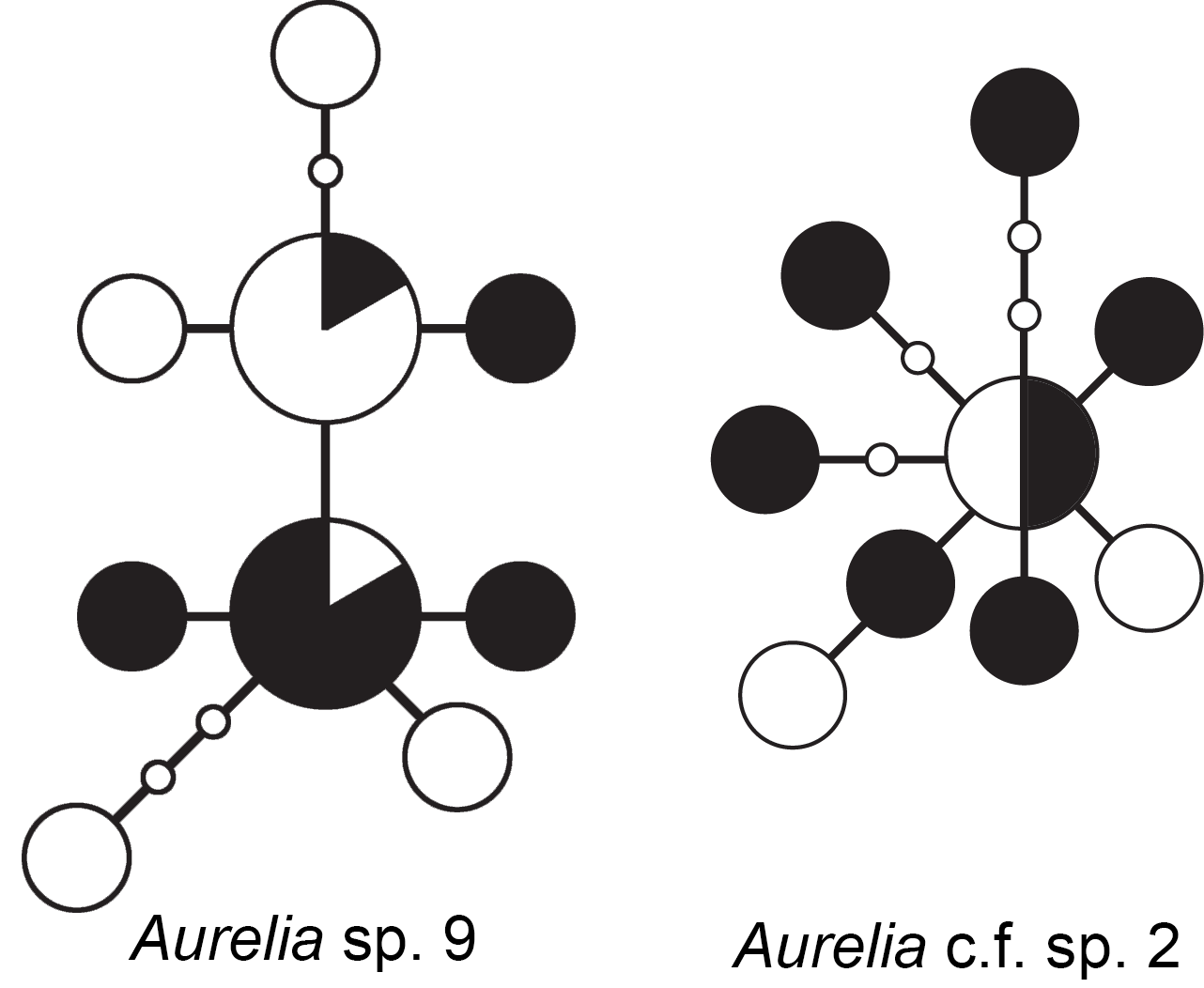


S1 Fig. Network tree based on *COI* for *Aurelia* sp. 9 and *Aurelia* c.f. sp. 2 in the Gulf of Mexico.

Different colors indicate different locations, with black representing the Northern Gulf of Mexico (Dauphin Island, AL) and white representing the Southeastern Gulf (Long Key, FL). Lines represent one mutational step and small black circles dots are inferred alleles that were not sampled. The area of each circle is proportional to the number of individuals sharing that particular allele, with the smallest circles representing a single individual.
