# Supplementary material for: Local versus Generalized Phenotypes in Two Sympatric Aurelia Species: Understanding Jellyfish Ecology Using Genetics and Morphometrics
Source: PLoS One. 2016 Jun 22;11(6):e0156588. doi: 10.1371/journal.pone.0156588 (PMC4917110; doi:10.1371/journal.pone.0156588)
Supplement: S2 Fig — (DOCX) [file pone.0156588.s002.docx]

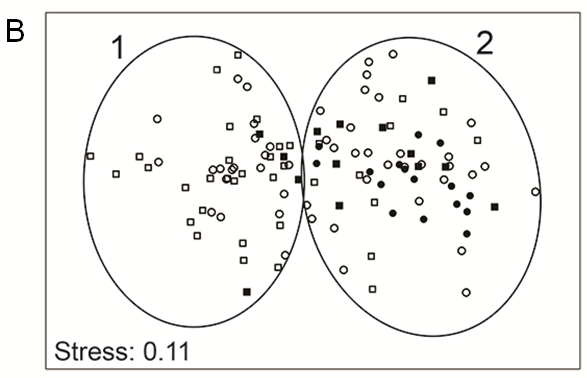

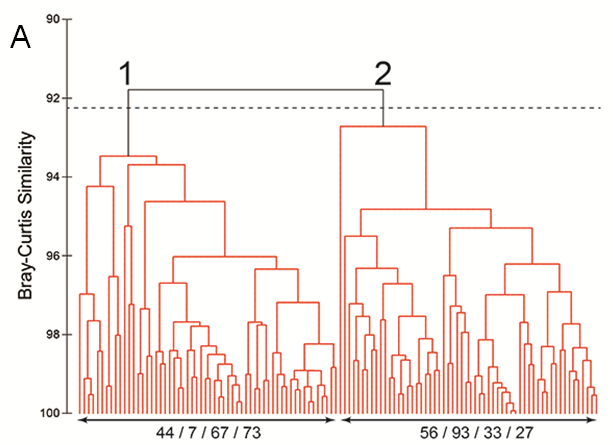


S2 Figure. Morphological variation in *Aurelia* from the Gulf of Mexico based on meristic features.

A: Cluster analysis. Numbers indicate main morphologically homogeneous clusters/groups (SIMPROF, α = 0.01). B. Multi-dimensional scaling analysis (MDS). Numbers correspond to main clusters obtained by Cluster analysis. Different symbols indicate different monophyletic *Aurelia* clades based on COI. Circles: *Aurelia* sp. 9 clade. Squaeres: *Aurelia* c.f. sp. 2. Solid symbols indicate molecular + morphological data, while open symbols indicate morphological data only. Numbers below clusters indicate the percentage of *Aurelia* sp.9 from CNGoM, *Aurelia* sp. 9 from SEGoM, *Aurelia* c.f. sp. 2 from CNGoM, and *Aurelia* c.f. sp. 2 from SEGoM, respectively, included in each cluster/group. Contours represent 95% confidence.
