# Supplementary material for: Local versus Generalized Phenotypes in Two Sympatric Aurelia Species: Understanding Jellyfish Ecology Using Genetics and Morphometrics
Source: PLoS One. 2016 Jun 22;11(6):e0156588. doi: 10.1371/journal.pone.0156588 (PMC4917110; doi:10.1371/journal.pone.0156588)
Supplement: S1 Table — (DOCX) [file pone.0156588.s003.docx]

S1 Table. List of sequences obtained from Genbank used in this study

| **Accession Number** | **Species** | **Location** |
| --- | --- | --- |
|  |  |  |
| Mitochondrial *COI* |  |  |
| AY903117 | *A. aurita* | Bosporus, Turkey |
| AY903118 | *A. aurita* | Gullmar Fjord, Sweden |
| AY903093 | *A. aurita* | Boston Harbor, U.S.A. |
| AY903085 | *Aurelia* sp. 1 | Los Angeles, California |
| AY903157 | *Aurelia* sp. 1 | New South Wales, Australia |
| AY903199 | *Aurelia* sp. 1 | Inland Sea, Japan |
| AY903120 | *Aurelia* sp. 2 | Cananeia, Brazil |
| AY903121 | *Aurelia* sp. 2 | Cananeia, Brazil |
| AY903119 | *Aurelia* sp. 2 | Cananeia, Brazil |
| AY903115 | *Aurelia* sp. 3 | Koror State, Palau |
| AY903096 | *Aurelia* sp. 3 | Koror State, Palau |
| AY903114 | *Aurelia* sp. 3 | Koror State, Palau |
| AY903137 | *Aurelia* sp. 4 | Oahu, Hawaii |
| AY903144 | *Aurelia* sp. 4 | Kakaban Island, Indonesia |
| AY903109 | *Aurelia* sp. 4 | Koror State, Palau |
| AY903123 | *Aurelia* sp. 5 | Lake Mljet, Croatia |
| AY903124 | *Aurelia* sp. 5 | Lake Mljet, Croatia |
| AY903125 | *Aurelia* sp. 5 | Lake Mljet, Croatia |
| AY903104 | *Aurelia* sp. 6 | Southwest Islands, Palau |
| AY903129 | *Aurelia* sp. 6 | New Britain, Papua New Guinea |
| AY903100 | *Aurelia* sp. 6 | Koror State, Palau |
| AY903139 | *Aurelia* sp. 7 | Tasmania, Australia |
| AY903140 | *Aurelia* sp. 7 | Tasmania, Australia |
| AY903141 | *Aurelia* sp. 7 | Tasmania, Australia |
| AY903134 | *Aurelia* sp. 8 | Bay of Ston, Croatia |
| AY903133 | *Aurelia* sp. 8 | North Adriatic |
| AY903135 | *Aurelia* sp. 8 | Bay of Ston, Croatia |
| AY903175 | *Aurelia* sp. 9 | Northern Gulf of Mexico, USA |
| AY903172 | *Aurelia* sp. 9 | Northern Gulf of Mexico, USA |
| AY903189 | *Aurelia limbata* | Hokkaido, Japan |
| AY903213 | *Aurelia* sp. 11 | Kwajalein, Marshall Islands |
| AY903068 | *Aurelia labiata* | Canada, British Columbia |
| AY903072 | *Aurelia labiata* | Canada, British Columbia |
| GQ120097 | *Phacellophora camtschatica* | Unknown |
|  |  |  |
| Nuclear *ITS-1* |  |  |
| AY935204 | *Aurelia* sp. 2 | Cananeia, Brazil |
| AY319850 | *Aurelia* sp. 2 | Cananeia, Brazil |
| AY319851 | *Aurelia* sp. 2 | Cananeia, Brazil |
| AY935216 | *Aurelia* sp. 9 | Northern Gulf of Mexico, USA |
