# Supplementary material for: Local versus Generalized Phenotypes in Two Sympatric Aurelia Species: Understanding Jellyfish Ecology Using Genetics and Morphometrics
Source: PLoS One. 2016 Jun 22;11(6):e0156588. doi: 10.1371/journal.pone.0156588 (PMC4917110; doi:10.1371/journal.pone.0156588)
Supplement: S2 Table — (DOCX) [file pone.0156588.s004.docx]

| ***ID*** | ***Continuous features*** |
| --- | --- |
| *f1* | Diameter of bell between tips of opposed rhopalia along perradial axis |
| *f2* | Mean length of oral arms taken from the base of the manubrium to the tip of oral arm |
| *f3* | Mean width of oral arms measured at half-length of oral arm |
| *f4* | Mean width of manubrium measured between the bases of opposing oral arms |
| *f5* | Mean length of manubrium calculated by subtracting bell height from medusa total height |
| *f6* | Mean distal gastric distance taken between the most distal points of opposing cavities |
| *f7* | Mean proximal gastric distance taken between the most proximal points of opposing cavities |
| *f8* | Size of gonads calculated by subtracting *f7* from *f8* |
| *f9* | Mean depth of rhopalar indentations taken as the distance from a tangent line across adjacent velar lobes to ring canal |
| *f10* | Mean depth of non-rhopalar indentations taken as the distance from a tangent line across adjacent velar lobes to ring canal |
| *f11* | Mean length of rhopalia measured from the base to the tip of rhopalar lobe |
| *f12* | Bell height measured at the center of the umbrella |
|  | ***Meristic features*** |
| *f13* | Mean number of originations of the gastravascular canal system per perradial quadrant |
| *f14* | Mean number of originations of the gastravascular canal system per interradial quadrant |
| *f15* | Mean number of originations of adradial canals |
| *f16* | Mean number of anastomoses per perradial quadrant |
| *f17* | Mean number of anastomoses per interradial quadrant |
| *f18* | Mean number of anastomoses of adradial canals |
| *f19* | Maximum number of branching points of the canal system within a perradial quadrant |
|  | ***Categorical features*** |
| *f20* | Color of gastric tissue (nominal): 1) no color, 2) light-pink/pink, 3) dark-pink/purple |
| *f21* | Color of gonadal tissue (nominal): 1) no color, 2) light-pink/pink, 3) dark-pink/purple |
| *f22* | Color of the bell (nominal): 1) no color, 2) light-pink/pink, 3) dark-pink/purple |
| *f23* | Color of bell margin (nominal): 1) no color, 2) light-pink/pink, 3) dark-pink/purple, 4) light brown |
| *f24* | Color of gastrovacular canals (nominal): 1) no color, 2) light-pink/pink, 3) dark-pink/purple |
| *f25* | Shape of the gonads (nominal): 1) u-shape, 2) horseshoe, 3) drop-like |
| *f26* | Degree of folding (i.e., "frilliness") of the oral arms (ordinal): from 1 to 5 at half-point intervals |
| *f27* | Degree of thickening of mesoglea surrounding sub-genital pore (ordinal): from 1 to 5 at half-point intervals |
| *f28* | Bell shape determined by the best-fit curve through measurements of bell height (nominal): 1) concave, 2) convex, 3) flat |

S2 Table. Morphological features recorded per medusa in this study
