# Supplementary material for: Local versus Generalized Phenotypes in Two Sympatric Aurelia Species: Understanding Jellyfish Ecology Using Genetics and Morphometrics
Source: PLoS One. 2016 Jun 22;11(6):e0156588. doi: 10.1371/journal.pone.0156588 (PMC4917110; doi:10.1371/journal.pone.0156588)
Supplement: S3 Table — (DOCX) [file pone.0156588.s005.docx]

S3 Table. Minimum pairwise genetic distances (Kimura 2-parameter) between *Aurelia* species/clades based on *COI* sequence data.

|  | 1 | 2 | 3 | 4 | 5 | 6 | 7 | 8 | 9 | 10 | 11 | 12 | 13 | 14 | 15 |
| --- | --- | --- | --- | --- | --- | --- | --- | --- | --- | --- | --- | --- | --- | --- | --- |
| 1 | **0.007** |  |  |  |  |  |  |  |  |  |  |  |  |  |  |
| 2 | 0.186 | **0.002** |  |  |  |  |  |  |  |  |  |  |  |  |  |
| 3 | 0.196 | 0.189 | **0.017** |  |  |  |  |  |  |  |  |  |  |  |  |
| 4 | 0.244 | 0.248 | 0.257 | **0** |  |  |  |  |  |  |  |  |  |  |  |
| 5 | 0.186 | 0.181 | 0.213 | 0.266 | **0.007** |  |  |  |  |  |  |  |  |  |  |
| 6 | 0.192 | 0.206 | 0.214 | 0.262 | 0.167 | **0.010** |  |  |  |  |  |  |  |  |  |
| 7 | 0.197 | 0.229 | 0.202 | 0.278 | 0.185 | 0.187 | **0.005** |  |  |  |  |  |  |  |  |
| 8 | 0.157 | 0.191 | 0.194 | 0.231 | 0.164 | 0.185 | 0.191 | **0.022** |  |  |  |  |  |  |  |
| 9 | 0.156 | 0.177 | 0.214 | 0.261 | 0.182 | 0.167 | 0.159 | 0.150 | **0** |  |  |  |  |  |  |
| 10 | 0.210 | 0.171 | 0.186 | 0.253 | 0.214 | 0.225 | 0.208 | 0.207 | 0.203 | **0.008** |  |  |  |  |  |
| 11 | 0.221 | 0.245 | 0.247 | 0.124 | 0.256 | 0.232 | 0.232 | 0.242 | 0.228 | 0.231 | **0.010** |  |  |  |  |
| 12 | 0.180 | 0.178 | 0.163 | 0.280 | 0.196 | 0.196 | 0.202 | 0.203 | 0.202 | 0.192 | 0.244 | **-** |  |  |  |
| 13 | 0.199 | 0.206 | 0.215 | 0.233 | 0.172 | 0.212 | 0.201 | 0.218 | 0.217 | 0.213 | 0.238 | 0.214 | **-** |  |  |
| 14 | 0.241 | 0.235 | 0.267 | 0.110 | 0.246 | 0.263 | 0.257 | 0.257 | 0.248 | 0.236 | 0.118 | 0.288 | 0.242 | **0.008** |  |
| 15 | 0.219 | 0.255 | 0.249 | 0.114 | 0.264 | 0.233 | 0.260 | 0.247 | 0.235 | 0.243 | 0.098 | 0.244 | 0.266 | 0.127 | **-** |

Values shown below diagonal are minimum pairwise genetic distances between species/clades. Values shown along diagonal (in bold) represent maximum pairwise genetic distance within species/clades, and are shown where multiple sequences were used in the analysis. Comparison important to species in the Gulf of Mexico are bound by rectangles. 1) *Aurelia aurita* (Turkey, Sweden, Boston-USA), 2) *Aurelia labiata* (British Columbia, Canada), 3) *Aurelia* sp. 1 (California-USA, New South Wales-Australia, Inland Sea, Japan), 4) *Aurelia* sp. 2 (Brazil), 5) *Aurelia* sp. 3 (Palau), 6) *Aurelia* sp. 4 (Hawaii-USA, Indonesia, Palau), 7) *Aurelia* sp. 5 (Mljet-Croatia), 8) *Aurelia* sp. 6 (Palau, New Guinea), 9) *Aurelia* sp. 7 (Tasmania-Australia), 10) *Aurelia* sp. 8 (Croatia, North Adriatic), 11) *Aurelia* sp. 9 (Gulf of Mexico-USA, This study), 12) *Aurelia limbata (Aurelia* sp. 10*;* Hokkaido-Japan), 13) *Aurelia sp. 11* (Kwajalein-Marshall Islands), 14) *Aurelia* c.f. sp. 2 (Gulf of Mexico-USA; this study), 15) *Aurelia* sp. DI’03-4 (this study).
