# Supplementary material for: Local versus Generalized Phenotypes in Two Sympatric Aurelia Species: Understanding Jellyfish Ecology Using Genetics and Morphometrics
Source: PLoS One. 2016 Jun 22;11(6):e0156588. doi: 10.1371/journal.pone.0156588 (PMC4917110; doi:10.1371/journal.pone.0156588)
Supplement: S4 Table — (DOCX) [file pone.0156588.s006.docx]

S4 Table. Summary of the AMOVA based on *COI* for *Aurelia* sp. 9 and *Aurelia* c.f. sp. 2 in the Gulf of Mexico.

| Species | Source of variation | df | Sum of Squares | Variance Components | Percentage of variation | F_st_ |
| --- | --- | --- | --- | --- | --- | --- |
| *Aurelia* sp. 9 | Between locations | 1 | 1.371 | 0.06038V_a_ | 6.47 | 0.065 |
|  | Within locations | 15 | 13.10 | 0.87333V_b_ | 93.53 |  |
|  | Total | 15 | 14.471 | 0.93371 |  |  |
| *Aurelia* c.f. sp. 2 | Between locations | 1 | 1.110 | -0.0253V_a_ | -1.95 | 0.683 |
|  | Within locations | 9 | 11.071 | 1.23016V_b_ | 101.95 |  |
|  | Total | 10 | 12.182 | 1.20663 |  |  |

CNGoM: Central Northern Gulf of Mexico, Dauphin Island AL. SEGoM: Southeastern Gulf of Mexico, Long Key FL.
