# Supplementary material for: Local versus Generalized Phenotypes in Two Sympatric Aurelia Species: Understanding Jellyfish Ecology Using Genetics and Morphometrics
Source: PLoS One. 2016 Jun 22;11(6):e0156588. doi: 10.1371/journal.pone.0156588 (PMC4917110; doi:10.1371/journal.pone.0156588)
Supplement: S5 Table — (DOCX) [file pone.0156588.s007.docx]

S5 Table. Variation in individual size-independent morphological features of *Aurelia* from the Gulf of Mexico.

| *Morphological features* | *Clade* | *Location* | *Clade x Location* |
| --- | --- | --- | --- |
| *Oral arm length (f2)* | F_1,117_= 378*** | F_1,117_= 10* | F_1,117_= 12* |
| *Oral arm width (f3)* | F_1,117_= 45** | F_1,117_= 43** | F_1,117_= 41** |
| *Manubrium width (f4)* | F_1,117_= 263*** | F_1,117_= 223*** | F_1,117_= 227*** |
| *Manubrium length (f5)* | F_1,117_= 515*** | F_1,117_= 20** | F_1,117_= 31** |
| *Distal gastric dist.(f6)* | F_1,117_= 310*** | F_1,117_= 63** | F_1,117_= 80** |
| *Prox. gastric dist. (f7)* | F_1,117_= 157*** | F_1,117_= 13* | F_1,117_= 10* |
| *Gonad size (f8)* | F_1,117_= 125*** | F_1,117_= 43** | F_1,117_= 71** |
| *Rhopalar ind. (f9)* | F_1,117_= 2.2 | F_1,117_= 0.5 | F_1,117_= 0.1 |
| *Non-rhopalar ind.(f10)* | F_1,117_= 680*** | F_1,117_= 83** | F_1,117_= 60** |
| *Rhopalium length (f11)* | F_1,117_= 3.1 | F_1,117_= 143** | F_1,117_= 231*** |
| *Bell height (f12)* | F_1,117_= 768*** | F_1,117_= 57** | F_1,117_= 76** |
| *Perradial origins (f13)* | F_1,117_= 2.1 | F_1,117_= 0.4 | F_1,117_= 1.3 |
| *Interradial origins (f14)* | F_1,117_= 1.1 | F_1,117_= 1.5 | F_1,117_= 0.2 |
| *Adradial origins (f15)* | F_1,117_= 2.1 | F_1,117_= 0.3 | F_1,117_= 1.2 |
| *Perradial anast. (f16)* | F_1,117_= 0.6 | F_1,117_= 0.5 | F_1,117_= 1.1 |
| *Interradial anast.(f17)* | F_1,117_= 0.3 | F_1,117_= 0.1 | F_1,117_= 0.4 |
| *Adradial anast. (f18)* | F_1,117_= 0.3 | F_1,117_= 0.1 | F_1,117_= 0.3 |
| *Branching points (f19)* | F_1,117_= 1.4 | F_1,117_= 0.3 | F_1,117_= 0.7 |
|  |  |  | *Groups* |
| *Gastric color (f20)* |  |  | χ^2^_(6)_ = 2.8 |
| *Gonad color (f21)* |  |  | χ^2^_(6)_ = 96.3** |
| *Bell color (f22)* |  |  | χ^2^_(6)_ = 96.5** |
| *Bell margin color (f23)* |  |  | χ^2^_(6)_ = 92.8** |
| *Canals color (f24)* |  |  | χ^2^_(6)_ = 2.2 |
| *Gonad shape (f25)* |  |  | χ^2^_(6)_ = 34.6** |
| *Oral arm folding (f26)* |  |  | H_3, 116_= 54.4** |
| *Mesoglea pore (f27)* |  |  | H_3, 116_= 63.4** |
| *Bell shape (f28)* |  |  | χ^2^_(6)_ = 3.7 |

*p<0.001, **p< 0.0001, *** p<0.00001

Variation in size-corrected continuous (*f2*-*f12*) and meristic (*f13*-*f19*) features were tested with a 2x2 factorial ANOVA with “Clade” (*Aurelia* sp. 9 and *Aurelia* c.f. sp. 2) and “Location” (Dauphin Island, AL -CNGoM- and Long Key, FL -SEGoM) as fixed factors. Variation in ordinal (*f26* and *f27*) and nominal categorical features (*f20*-*f25*, *f28*) was tested among four pre-determined groups (I: *Aurelia* sp.9 from CNGoM, II: *Aurelia* sp9 from SEGoM, III*: Aurelia* sp.12 from CNGoM, IV: *Aurelia* sp.12 from SEGoM) by Kruskal-Wallis andχ^2^tests, respectively, using a one-way approach. See Material and Methods section for details.
